# A Comparison of the Adverse Effects and Utility of Different Monoclonal Antibodies for SARS-CoV-2: A Retrospective Cohort Study

**DOI:** 10.7759/cureus.43094

**Published:** 2023-08-07

**Authors:** Brandon W Knopp, Hannah Z Weiss, Samer Fahmy, Evan Goldstein, Jeniel Parmar

**Affiliations:** 1 Department of Emergency Medicine, Florida Atlantic University Charles E. Schmidt College of Medicine, Boca Raton, USA; 2 Department of Emergency Medicine, Boca Raton Regional Hospital, Boca Raton, USA

**Keywords:** public health, hospitalization rate, emergency medicine, infectious disease, monoclonal antibodies, covid 19

## Abstract

Introduction

Multiple monoclonal antibody (mAb) treatments have been developed to combat the growing number of severe acute respiratory syndrome coronavirus 2 (SARS-CoV-2) strains. These treatments have been shown to be effective in reducing the risk of hospitalization and death from SARS-CoV-2 infection with a low risk of adverse effects; however, more data is required to evaluate the comparative efficacy of mAbs. The primary objective of this study is to describe the hospitalization rate, length of stay (LOS), and mortality rate in SARS-CoV-2 patients treated with four different mAb treatments, including bamlanivimab plus etesevimab, casirivimab plus imdevimab, sotrovimab, and bebtelovimab.

Methods

A retrospective chart review and prospective phone surveys of SARS-CoV-2 patients treated with mAbs in a 400-bed tertiary, suburban medical center were conducted between June 2020 and April 2022. Eligibility criteria for mAbs included non-hospitalized patients over the age of 18 with less than 10 days of SARS-CoV-2 symptoms and no oxygen requirement on emergency department (ED) admission. Data were collected from the retrospective chart review and subjective patient surveys. A chi-squared test was used. Significance was assessed at p < 0.05.

Results

The study population included 3249 patients, with 1537 males and 1712 females and an average age of 62.48 ± 17.54 years. Five hundred forty-two patients received bamlanivimab plus etesevimab; 849 received bebtelovimab; 1577 received casirivimab plus imdevimab; and 281 received sotrovimab. The overall hospitalization rate was 1.0%, and the mortality rate was 0.2% following mAb treatment. The hospitalization rate was greatest among patients administered Sotrovimab (2.1%) and least among patients administered Bebtelovimab (0.1%) (p = 0.010). 2.4% of patients who were discharged from the ED after receiving one of the four mAbs returned within 30 days with SARS-CoV-2 symptoms. The average length of stay was 4.75 ± 4.56 days, with no significant differences between the mAbs. The provider-reported adverse event rate was 2.2%, with significant differences in adverse event rates between mAbs. Bamlanivimab-etesevimab was associated with the highest adverse event rate (4.6%), and sotrovimab was associated with the lowest adverse event rate (1.4%) (p < 0.001).

Conclusion

This study shows a low hospitalization and mortality rate following mAb infusion in patients with mild and moderate COVID-19. However, there were significant differences in hospitalization and mortality among patients receiving each of the four mAb treatments. There was a high degree of patient-reported symptom improvement, and adverse reactions were reported in only 2.2% of patients with no severe reactions. Multiple monoclonal antibody treatments are not effective as monotherapy; however, this study shows the potential benefits of including a mAb infusion as part of a SARS-CoV-2 treatment plan.

## Introduction

The COVID-19 pandemic, caused by the severe acute respiratory syndrome coronavirus 2 (SARS-CoV-2), has been the most globally impactful health crisis since the influenza pandemic of 1918 [1]. A variety of treatments and preventative measures have been introduced to combat SARS-CoV-2 and limit the cytokine-predominant inflammatory response, also called a cytokine storm, which mediates much of the pathology of SARS-CoV-2 [1-3]. The therapeutic options include antiviral drugs, anti-inflammatory drugs, immunomodulatory agents, vaccines, and four different monoclonal antibodies (mAbs) [1,4]. These treatments are useful at different stages of the disease or, in the case of the various vaccines available, before infection to mitigate the risk of severe infection [5].

Monoclonal antibodies have been developed to treat malignancies, autoimmune conditions, infectious diseases such as Ebola, and post-transplant immunosuppression [6-8]. The development of mAbs as an effective treatment tool over the last several decades has generated significant interest in developing mAbs targeted toward SARS-CoV-2 [9,10]. Monoclonal antibodies have been proposed as a prophylactic and therapeutic treatment for non-hospitalized SARS-CoV-2 patients with mild to moderate symptoms to prevent progression to more severe disease, which may necessitate hospitalization [7,11]. Monoclonal antibodies treat active SARS-CoV-2 by targeting the spike protein central to the infectivity of the SARS-CoV-2 virus [7]. This activity reduces viral entry into cells and the subsequent cytokine storm, which occurs via the release of cytokines including tumor necrosis factor-alpha (TNF-α), interleukin (IL)-1, IL-6, granulocyte-macrophage colony-stimulating factor (GM-CSF), and interferon‐gamma (IFN‐γ) [7]. The weakened immune reaction and decreased inflammatory state caused by a mAb infusion reduce disease severity and may improve clinical outcomes [12,13].

Monoclonal antibodies have been shown in multiple studies to have a risk for adverse reactions ranging from chills, flushing, fever, and rash to severe hypotension, anaphylaxis, and arrhythmias [14-16]. However, the potential benefits of mAb infusions may outweigh the risk of potential adverse reactions in populations most at risk for severe SARS-CoV-2 infection, such as those with preexisting diabetes, cancer, obesity, chronic lung disease, cardiovascular disease, chronic kidney disease, an immunocompromised status, a smoking history, and those aged ≥65 years [2,17-19]. In the early stage of the disease, before the onset of severe symptoms, mAbs have been shown to attenuate the immune response to SARS-CoV-2 and reduce the severity of infection [2,20-23]. Monoclonal antibody treatments are formulated to target specific variants of SARS-CoV-2 and have been shown to have varying efficacy against its viral strains. Multiple monoclonal antibody variations are available to treat different SARS-CoV-2 strains, including bamlanivimab plus etesevimab, casirivimab plus imdevimab, sotrovimab, and bebtelovimab. Eligibility criteria for mAbs included non-hospitalized patients with less than 10 days of SARS-CoV-2 symptoms and no oxygen requirement on emergency department (ED) admission.

Clinical trials such as the Blocking Viral Attachment and Cell Entry with SARS-CoV-2 Neutralizing Antibodies (BLAZE) trials and the COVID-19 Monoclonal Antibody Efficacy Trial-Intent to Care Early (COMET-ICE) trial have shown that bamlanivimab with or without etesevimab (BLAZE trials), bebtelovimab (BLAZE trials), sotrovimab (COMET-ICE trial), and casirivimab plus imdevimab are effective in reducing viral load and reducing the risk of hospitalization and death [21,24]. Likewise, adverse reactions in patients receiving casirivimab plus imdevimab, bamlanivimab with or without etesevimab, sotrovimab, and bebtelovimab were similar to those in patients receiving placebo [10,22,24]. These trials and other studies have provided information on clinical results with mAb use; however, more data are needed to understand the efficacy of these mAbs in the treatment of SARS-CoV-2 and describe the rate of adverse reactions. This study investigates the use of mAbs to treat SARS-CoV-2 patients and examines the clinical course, adverse effects, and subjective improvements following mAb infusion.

Primary objective

The primary objective of this study is to describe the hospitalization rate, length of stay (LOS), adverse effects, and mortality rate in SARS-CoV-2 patients treated with the four different monoclonal antibody treatments. 

Secondary objective

The secondary objective of this study is to evaluate the subjective improvement and patient-reported adverse effects in SARS-CoV-2 patients treated with the four different monoclonal antibody treatments.

## Materials and methods

Study design and setting

Retrospective chart review and prospective phone surveys of all SARS-CoV-2 patients treated with four different monoclonal antibodies between June 2020 and April 2022 were conducted at Baptist Health Boca Raton Regional Hospital, a 400-bed tertiary, suburban medical center in Boca Raton, Florida. Patients administered monoclonal antibodies met the CDC criteria upon ED admission and gave informed consent to treatment. Inclusion criteria were SARS-CoV-2 patients treated with monoclonal antibodies and all patients 18 years or older. There were no exclusion criteria. A positive polymerase chain reaction (PCR) test for SARS-CoV-2 taken in the ED was required for a diagnosis of SARS-CoV-2. Patients who were treated with monoclonal antibody infusions for SARS-CoV-2 infection at the Boca Raton Regional Hospital were identified by requesting a query from the Boca Raton Regional Hospital Information Technology (IT) department. Subjects identified from the query were reviewed by the Investigators for inclusion and exclusion criteria during subsequent chart review. Ethics approval was granted by the Baptist Health South Florida Institutional Review Board (approval number: 1899481-1). 

Data collection

Data collected from the retrospective chart review and subjective patient surveys included: symptoms upon initial presentation, vaccination status, age, sex, the timing of administration, comorbidities, immunocompetency, complication rate, hospitalization rate, mortality rate, hospital LOS, adverse effects, subjective improvements, concurrent treatments, and other pertinent observations following monoclonal antibody administration. All patients admitted to the ED were asked about a standardized set of symptoms. The mAb adverse effects were classified as mild or severe by symptoms. Mild adverse effects included symptoms such as chills, fever, mild hypotension, and flushing, while severe adverse effects involved severe hypotension, anaphylaxis, or cardiac dysfunction [14]. Charts were queried for subsequent hospital visits or death within 30 days after the infusion date. Eligible patients were called and administered a verbal telephone survey by trained research assistants. Informed consent was obtained at the time of the telephone contact with the subject, and the survey was not administered to any subject who indicated they did not wish to participate in the survey portion of the study. The survey consisted of three questions and asked about symptom improvement, adverse effects, and vaccination history.

Statistical analysis

Descriptive statistics were used to characterize patient demographics and outcome variables. Quantitative data were expressed as the mean + standard error of the mean (SEM), and nominal data was expressed as a percentage. A chi-squared test was used. Significance was assessed at p < 0.05. The analysis was conducted using the Statistical Analysis System (SAS) (SAS Institute Inc., Cary, NC, United States).

## Results

Patient characteristics

Table 1 compares the characteristics of the study participants.

**Table 1:** Patient demographics, vaccination status, and medical conditions

	Bamlanivimab-Etesevimab (N=542)	Bebtelovimab (N=849)	Casirivimab-Imdevimab (N=1577)	Sotrovimab (N=281)	Total (N=3249)	P-Value
Age (years)						
Mean (SD)	64.83 (15.24)	68.78 (15.43)	57.13 (18.19)	69.00 (14.16)	62.48 (17.54)	< 0.001
Range	16.00 - 98.00	14.00 - 103.00	13.00 - 99.00	23.00 - 104.00	13.00 - 104.00	
BMI (kg/m2)						
Mean (SD)	34.39 (6.96)	27.69 (6.89)	29.81 (7.13)	27.96 (4.17)	30.08 (7.25)	< 0.001
Range	20.00 - 56.00	16.00 - 50.00	17.00 - 60.00	20.00 - 40.00	16.00 - 60.00	
Participants with BMI Over 30 kg/m2						
No	20 (20.6%)	1 (2.5%)	12 (9.0%)	0 (0.0%)	33 (11.8%)	0.005
Yes	77 (79.4%)	39 (97.5%)	122 (91.0%)	9 (100.0%)	247 (88.2%)	
Current Smoker						
No	520 (95.9%)	827 (97.4%)	1499 (95.1%)	271 (96.4%)	3117 (95.9%)	0.045
Yes	22 (4.1%)	22 (2.6%)	78 (4.9%)	10 (3.6%)	132 (4.1%)	
Past Smoker						
No	460 (84.9%)	730 (86.0%)	1475 (93.5%)	257 (91.5%)	2922 (89.9%)	< 0.001
Yes	82 (15.1%)	119 (14.0%)	102 (6.5%)	24 (8.5%)	327 (10.1%)	
Female						
No	272 (50.2%)	384 (45.2%)	747 (47.4%)	134 (47.7%)	1537 (47.3%)	0.349
Yes	270 (49.8%)	465 (54.8%)	830 (52.6%)	147 (52.3%)	1712 (52.7%)	
Race						
White	443 (81.7%)	677 (80.0%)	1170 (75.1%)	217 (78.1%)	2507 (77.8%)	< 0.001
Black	20 (3.7%)	11 (1.3%)	65 (4.2%)	2 (0.7%)	98 (3.0%)	
Asian	0 (0.0%)	4 (0.5%)	5 (0.3%)	0 (0.0%)	9 (0.3%)	
Other	79 (14.6%)	154 (18.2%)	318 (20.4%)	59 (21.2%)	610 (18.9%)	
Hispanic						
No	513 (94.6%)	819 (96.5%)	1444 (91.6%)	270 (96.1%)	3046 (93.8%)	< 0.001
Yes	29 (5.4%)	30 (3.5%)	133 (8.4%)	11 (3.9%)	203 (6.2%)	
Vaccinated						
Yes	55 (10.1%)	761 (89.6%)	928 (58.8%)	265 (94.3%)	2009 (61.8%)	< 0.001
No	487 (89.9%)	88 (10.4%)	649 (41.2%)	16 (5.7%)	1240 (38.2%)	
First and Second Vaccine Doses Only						
No	512 (94.5%)	717 (84.5%)	858 (54.4%)	241 (85.8%)	2328 (71.7%)	< 0.001
Yes	30 (5.5%)	132 (15.5%)	719 (45.6%)	40 (14.2%)	921 (28.3%)	
First and Second Vaccine Doses and Booster						
No	526 (97.0%)	357 (42.0%)	1461 (92.6%)	62 (22.1%)	2406 (74.1%)	< 0.001
Yes	16 (3.0%)	492 (58.0%)	116 (7.4%)	219 (77.9%)	843 (25.9%)	
Two Vaccine Doses and Two Booster Doses						
No	542 (100.0%)	723 (85.2%)	1577 (100.0%)	281 (100.0%)	3123 (96.1%)	< 0.001
Yes	0 (0.0%)	126 (14.8%)	0 (0.0%)	0 (0.0%)	126 (3.9%)	
Moderna						
No	530 (97.8%)	593 (69.8%)	1311 (83.1%)	190 (67.6%)	2624 (80.8%)	< 0.001
Yes	12 (2.2%)	256 (30.2%)	266 (16.9%)	91 (32.4%)	625 (19.2%)	
Pfizer						
No	511 (94.3%)	385 (45.3%)	1005 (63.7%)	120 (42.7%)	2021 (62.2%)	< 0.001
Yes	31 (5.7%)	464 (54.7%)	572 (36.3%)	161 (57.3%)	1228 (37.8%)	
Johnson & Johnson						
No	542 (100.0%)	830 (97.8%)	1509 (95.7%)	271 (96.4%)	3152 (97.0%)	< 0.001
Yes	0 (0.0%)	19 (2.2%)	68 (4.3%)	10 (3.6%)	97 (3.0%)	
Other/Combination of Vaccines Received						
No	542 (100.0%)	813 (95.8%)	1566 (99.3%)	279 (99.3%)	3200 (98.5%)	< 0.001
Yes	0 (0.0%)	36 (4.2%)	11 (0.7%)	2 (0.7%)	49 (1.5%)	
History of Cancer						
No	488 (90.0%)	723 (85.2%)	1420 (90.0%)	222 (79.0%)	2853 (87.8%)	< 0.001
Yes	54 (10.0%)	126 (14.8%)	157 (10.0%)	59 (21.0%)	396 (12.2%)	
Diabetes Mellitus						
No	441 (81.4%)	752 (88.6%)	1447 (91.8%)	244 (86.8%)	2884 (88.8%)	< 0.001
Yes	101 (18.6%)	97 (11.4%)	130 (8.2%)	37 (13.2%)	365 (11.2%)	
Hypertension						
No	295 (54.4%)	534 (62.9%)	1188 (75.3%)	198 (70.5%)	2215 (68.2%)	< 0.001
Yes	247 (45.6%)	315 (37.1%)	389 (24.7%)	83 (29.5%)	1034 (31.8%)	
Hyperlipidemia						
No	410 (75.6%)	583 (68.7%)	1340 (85.0%)	214 (76.2%)	2547 (78.4%)	< 0.001
Yes	132 (24.4%)	266 (31.3%)	237 (15.0%)	67 (23.8%)	702 (21.6%)	
Coronary Artery Disease						
No	458 (84.5%)	741 (87.3%)	1467 (93.0%)	239 (85.1%)	2905 (89.4%)	< 0.001
Yes	84 (15.5%)	108 (12.7%)	110 (7.0%)	42 (14.9%)	344 (10.6%)	
Dementia						
No	534 (98.5%)	841 (99.1%)	1557 (98.7%)	280 (99.6%)	3212 (98.9%)	0.457
Yes	8 (1.5%)	8 (0.9%)	20 (1.3%)	1 (0.4%)	37 (1.1%)	
Parkinson’s Disease						
No	541 (99.8%)	840 (98.9%)	1574 (99.8%)	279 (99.3%)	3234 (99.5%)	0.015
Yes	1 (0.2%)	9 (1.1%)	3 (0.2%)	2 (0.7%)	15 (0.5%)	
Stroke						
No	526 (97.0%)	815 (96.0%)	1552 (98.4%)	280 (99.6%)	3173 (97.7%)	< 0.001
Yes	16 (3.0%)	34 (4.0%)	25 (1.6%)	1 (0.4%)	76 (2.3%)	
Asthma						
No	502 (92.6%)	785 (92.5%)	1462 (92.7%)	260 (92.5%)	3009 (92.6%)	0.997
Yes	40 (7.4%)	64 (7.5%)	115 (7.3%)	21 (7.5%)	240 (7.4%)	
Chronic Obstructive Pulmonary Disease (COPD)						
No	515 (95.0%)	810 (95.4%)	1526 (96.8%)	267 (95.0%)	3118 (96.0%)	0.157
Yes	27 (5.0%)	39 (4.6%)	51 (3.2%)	14 (5.0%)	131 (4.0%)	
Other-Cause Immunodeficiency						
No	515 (95.0%)	836 (98.5%)	1545 (98.0%)	265 (94.3%)	3161 (97.3%)	< 0.001
Yes	27 (5.0%)	13 (1.5%)	32 (2.0%)	16 (5.7%)	88 (2.7%)	
Systemic Inflammatory Disease						
No	500 (92.3%)	783 (92.2%)	1505 (95.4%)	242 (86.1%)	3030 (93.3%)	< 0.001
Yes	42 (7.7%)	66 (7.8%)	72 (4.6%)	39 (13.9%)	219 (6.7%)	

The study population included 3249 patients, with 1537 males and 1712 females and an average age of 62.48 ± 17.54 years. Minor differences seen in patient populations with each mAb are coincidental. Twenty-five patients (0.10%) declined to report their race, and body mass index (BMI) was reported for only 450 patients (13.9%). The majority of patients were vaccinated at least once prior to ED presentation (61.8%), with significant differences in vaccination rates between patients given different mAbs due to the timeframe during which each mAb was given (p < 0.001). No significant difference was found between hospitalization and the total number of vaccinations received (P = 0.4075). The most frequent pre-existing conditions were hypertension (31.8%), hyperlipidemia (21.6%), a history of cancer (12.2%), diabetes mellitus (11.2%), and coronary artery disease (10.6%).

Presentation 

Table 2 details patient symptoms, presentation, and treatments administered.

**Table 2:** Symptoms on presentation SARS-CoV-2: severe acute respiratory syndrome coronavirus 2; ED: emergency department; SD: standard deviation

	Bamlanivimab-Etesevimab (N=542)	Bebtelovimab (N=849)	Casirivimab-Imdevimab (N=1577)	Sotrovimab (N=281)	Total (N=3249)	P-Value
Asymptomatic						
No	524 (96.7%)	814 (95.9%)	1453 (92.1%)	266 (94.7%)	3057 (94.1%)	< 0.001
Yes	18 (3.3%)	35 (4.1%)	124 (7.9%)	15 (5.3%)	192 (5.9%)	
Fever						
No	230 (42.4%)	556 (65.5%)	774 (49.1%)	187 (66.5%)	1747 (53.8%)	< 0.001
Yes	312 (57.6%)	293 (34.5%)	803 (50.9%)	94 (33.5%)	1502 (46.2%)	
Cough						
No	102 (18.8%)	134 (15.8%)	451 (28.6%)	62 (22.1%)	749 (23.1%)	< 0.001
Yes	440 (81.2%)	715 (84.2%)	1126 (71.4%)	219 (77.9%)	2500 (76.9%)	
Body Ache						
No	183 (33.8%)	479 (56.4%)	773 (49.0%)	147 (52.3%)	1582 (48.7%)	< 0.001
Yes	359 (66.2%)	370 (43.6%)	804 (51.0%)	134 (47.7%)	1667 (51.3%)	
Rash						
No	542 (100.0%)	849 (100.0%)	1575 (99.9%)	281 (100.0%)	3247 (99.9%)	0.548
Yes	0 (0.0%)	0 (0.0%)	2 (0.1%)	0 (0.0%)	2 (0.1%)	
Shortness of Breath						
No	356 (65.7%)	805 (94.8%)	1271 (80.6%)	259 (92.2%)	2691 (82.8%)	< 0.001
Yes	186 (34.3%)	44 (5.2%)	306 (19.4%)	22 (7.8%)	558 (17.2%)	
Headache						
No	411 (75.8%)	701 (82.6%)	805 (51.0%)	193 (68.7%)	2110 (64.9%)	< 0.001
Yes	131 (24.2%)	148 (17.4%)	772 (49.0%)	88 (31.3%)	1139 (35.1%)	
Nausea or Vomiting						
No	423 (78.0%)	798 (94.0%)	1316 (83.4%)	250 (89.0%)	2787 (85.8%)	< 0.001
Yes	119 (22.0%)	51 (6.0%)	261 (16.6%)	31 (11.0%)	462 (14.2%)	
Weakness or Malaise						
No	498 (91.9%)	749 (88.2%)	1547 (98.1%)	270 (96.1%)	3064 (94.3%)	< 0.001
Yes	44 (8.1%)	100 (11.8%)	30 (1.9%)	11 (3.9%)	185 (5.7%)	
Fatigue						
No	399 (73.6%)	609 (71.7%)	648 (41.1%)	146 (52.0%)	1802 (55.5%)	< 0.001
Yes	143 (26.4%)	240 (28.3%)	929 (58.9%)	135 (48.0%)	1447 (44.5%)	
Diarrhea						
No	405 (74.7%)	783 (92.2%)	1292 (81.9%)	249 (88.6%)	2729 (84.0%)	< 0.001
Yes	137 (25.3%)	66 (7.8%)	285 (18.1%)	32 (11.4%)	520 (16.0%)	
Syncope						
No	542 (100.0%)	848 (99.9%)	1577 (100.0%)	281 (100.0%)	3248 (100.0%)	0.419
Yes	0 (0.0%)	1 (0.1%)	0 (0.0%)	0 (0.0%)	1 (0.0%)	
Dizziness						
No	542 (100.0%)	849 (100.0%)	1576 (99.9%)	281 (100.0%)	3248 (100.0%)	0.787
Yes	0 (0.0%)	0 (0.0%)	1 (0.1%)	0 (0.0%)	1 (0.0%)	
Days with SARS-CoV-2 Symptoms or Positive Test Before ED Presentation						
Mean (SD)	5.16 (2.67)	3.97 (1.69)	4.85 (2.79)	4.34 (2.95)	4.63 (2.58)	< 0.001
Range	0.00 - 35.00	0.00 - 20.00	0.00 - 38.00	0.00 - 42.00	0.00 - 42.00	

While the most common presenting symptoms were cough (76.9%), body ache (51.3%), fever (46.2%), fatigue (44.5%), and headache (35.1%), there were 194 patients (6.0%) who were asymptomatic on presentation. Only 17.2% of patients presented to the ED with difficulty breathing or shortness of breath. Patients reported having a positive SARS-CoV-2 test or SARS-CoV-2 symptoms, whichever came first, 4.63 ± 2.58 days (range: 0-42 days) prior to ED presentation on average. There were 188 patients (5.8%) who received a steroid bolus and 293 patients (9.0%) who received a vitamin infusion. A minority of patients (2.6%) were given additional antibiotic or antiviral medications used in the treatment of SARS-CoV-2, including azithromycin, acyclovir, valacyclovir, remdesivir, paxlovid, hydroxychloroquine, or ivermectin.

Primary objective

The hospitalization rate, mortality rate, and subsequent ED visits are reported in Table 3.

**Table 3:** Hospitalization rate, mortality rate, and repeat ED visit(s) SARS‑CoV‑2: severe acute respiratory syndrome coronavirus 2; ED: emergency department; SD: standard deviation

	Bamlanivimab-Etesevimab (N=542)	Bebtelovimab (N=849)	Casirivimab-Imdevimab (N=1577)	Sotrovimab (N=281)	Total (N=3249)	P-Value
Hospitalized						
No	535 (98.7%)	848 (99.9%)	1559 (98.9%)	275 (97.9%)	3217 (99.0%)	0.010
Yes	7 (1.3%)	1 (0.1%)	18 (1.1%)	6 (2.1%)	32 (1.0%)	
Returned to the ED due to SARS-CoV-2 Symptoms						
No	528 (97.4%)	841 (99.1%)	1526 (96.8%)	275 (97.9%)	3170 (97.6%)	0.006
Yes	14 (2.6%)	8 (0.9%)	51 (3.2%)	6 (2.1%)	79 (2.4%)	
Days to ED Return						
Mean (SD)	3.29 (1.60)	3.63 (2.45)	4.61 (4.85)	4.80 (4.87)	4.36 (4.35)	0.842
Range	1.00 - 6.00	1.00 - 8.00	0.00 - 29.00	0.00 - 12.00	0.00 - 29.00	
No Oxygen Given						
No	536 (98.9%)	848 (99.9%)	1555 (98.6%)	275 (97.9%)	3214 (98.9%)	0.008
Yes	6 (1.1%)	1 (0.1%)	22 (1.4%)	6 (2.1%)	35 (1.1%)	
Low-Flow Oxygen						
No	537 (99.1%)	847 (99.8%)	1561 (99.0%)	279 (99.3%)	3224 (99.2%)	0.204
Yes	5 (0.9%)	2 (0.2%)	16 (1.0%)	2 (0.7%)	25 (0.8%)	
High-Flow Oxygen						
No	538 (99.3%)	849 (100.0%)	1574 (99.8%)	281 (100.0%)	3242 (99.8%)	0.025
Yes	4 (0.7%)	0 (0.0%)	3 (0.2%)	0 (0.0%)	7 (0.2%)	
Mortality						
No	539 (99.4%)	849 (100.0%)	1572 (99.7%)	281 (100.0%)	3241 (99.8%)	0.158
Yes	3 (0.6%)	0 (0.0%)	5 (0.3%)	0 (0.0%)	8 (0.2%)	
Length of Stay (Days)						
Mean (SD)	5.53 (7.12)	8.33 (1.53)	4.38 (3.64)	3.71 (2.56)	4.75 (4.56)	0.411
Range	1.00 - 28.00	7.00 - 10.00	1.00 - 20.00	1.00 - 9.00	1.00 - 28.00	

Thirty-two patients (1.0%) were hospitalized following the initial ED presentation, with an additional 39 patients (1.2%) who were discharged from the ED initially but later admitted. There were 79 total patients (2.4%) who were discharged from the ED after receiving a mAb infusion and returned within 30 days with SARS-CoV-2 symptoms. Patients hospitalized after being discharged following the initial ED visit returned 4.36 ± 4.35 days later (range: 0-29 days). Patients hospitalized following the initial or subsequent ED visit stayed for 4.75 ± 4.56 days (range: 1-28 days). A total of 10 patients in the study population died of SARS-CoV-2-related complications, three (0.1%) following the initial hospitalization and seven (0.2%) after presenting to the ED a second time. No patients were intubated, and only 32 (1.0%) were given oxygen, 25 (0.8%) with low-flow oxygen, and 7 (0.2%) with high-flow oxygen.

Secondary objective

Table 4 reports adverse reactions following mAb administration.

**Table 4:** Adverse reactions following monoclonal antibody administration SD: standard deviation

	Bamlanivimab-Etesevimab (N=542)	Bebtelovimab (N=849)	Casirivimab-Imdevimab (N=1577)	Sotrovimab (N=281)	Total (N=3249)	P-Value
Adverse Reaction						
No	517 (95.4%)	835 (98.4%)	1547 (98.1%)	277 (98.6%)	3176 (97.8%)	< 0.001
Yes	25 (4.6%)	14 (1.6%)	30 (1.9%)	4 (1.4%)	73 (2.2%)	
Time to Adverse Reaction (Hours)						
Mean (SD)	0.56 (1.40)	0.09 (0.30)	4.81 (12.98)	0.00 (0.00)	2.17 (8.60)	0.468
Range	0.00 - 4.00	0.00 - 1.00	0.00 - 48.00	0.00 - 0.00	0.00 - 48.00	
Fever						
No	542 (100.0%)	848 (99.9%)	1574 (99.8%)	281 (100.0%)	3245 (99.9%)	0.661
Yes	0 (0.0%)	1 (0.1%)	3 (0.2%)	0 (0.0%)	4 (0.1%)	
Rash						
No	542 (100.0%)	849 (100.0%)	1575 (99.9%)	280 (99.6%)	3246 (99.9%)	0.307
Yes	0 (0.0%)	0 (0.0%)	2 (0.1%)	1 (0.4%)	4 (0.1%)	
Difficulty Breathing or Shortness of Breath						
No	541 (99.8%)	847 (99.8%)	1573 (99.7%)	280 (99.6%)	3241 (99.8%)	0.973
Yes	1 (0.2%)	2 (0.2%)	4 (0.3%)	1 (0.4%)	8 (0.2%)	
Nausea or Vomiting						
No	538 (99.3%)	847 (99.8%)	1574 (99.8%)	281 (100.0%)	3240 (99.7%)	0.143
Yes	4 (0.7%)	2 (0.2%)	3 (0.2%)	0 (0.0%)	9 (0.3%)	
Mild Hypotension						
No	538 (99.3%)	847 (99.8%)	1573 (99.7%)	281 (100.0%)	3239 (99.7%)	0.218
Yes	4 (0.7%)	2 (0.2%)	4 (0.3%)	0 (0.0%)	10 (0.3%)	
Lightheadedness or Dizziness						
No	539 (99.4%)	843 (99.3%)	1572 (99.7%)	281 (100.0%)	3235 (99.6%)	0.336
Yes	3 (0.6%)	6 (0.7%)	5 (0.3%)	0 (0.0%)	14 (0.4%)	
Weakness						
No	541 (99.8%)	849 (100.0%)	1575 (99.9%)	281 (100.0%)	3246 (99.9%)	0.627
Yes	1 (0.2%)	0 (0.0%)	2 (0.1%)	0 (0.0%)	3 (0.1%)	
Back Pain or Back Spasms						
No	542 (100.0%)	848 (99.9%)	1577 (100.0%)	280 (99.6%)	3247 (99.9%)	0.127
Yes	0 (0.0%)	1 (0.1%)	0 (0.0%)	1 (0.4%)	2 (0.1%)	
Facial Flushing						
No	542 (100.0%)	847 (99.8%)	1576 (99.9%)	281 (100.0%)	3246 (99.9%)	0.425
Yes	0 (0.0%)	2 (0.2%)	1 (0.1%)	0 (0.0%)	3 (0.1%)	
Diaphoresis						
No	541 (99.8%)	849 (100.0%)	1574 (99.8%)	281 (100.0%)	3245 (99.9%)	0.545
Yes	1 (0.2%)	0 (0.0%)	3 (0.2%)	0 (0.0%)	4 (0.1%)	
Tachycardia						
No	542 (100.0%)	847 (99.8%)	1576 (99.9%)	281 (100.0%)	3246 (99.9%)	0.425
Yes	0 (0.0%)	2 (0.2%)	1 (0.1%)	0 (0.0%)	3 (0.1%)	
Allergic Reaction						
No	541 (99.8%)	848 (99.9%)	1572 (99.7%)	280 (99.6%)	3241 (99.8%)	0.774
Yes	1 (0.2%)	1 (0.1%)	5 (0.3%)	1 (0.4%)	8 (0.2%)	

There were 73 patients (2.2%) who experienced adverse reactions within 2.17 ± 8.60 hours following mAb administration. No severe adverse reactions were reported, and all patients experiencing an adverse reaction such as mild hypotension, lightheadedness, or dizziness improved clinically following the fluid bolus and/or temporarily halting the transfusion. The greatest frequency of adverse reactions occurred with bamlanivimab-etesevimab (4.6%), and the lowest frequency of adverse reactions occurred with sotrovimab (1.4%) (p < 0.001). The most common adverse reactions were dizziness (0.3%), mild hypotension (0.3%), nausea/vomiting (0.3%), and shortness of breath (SOB) (0.2%). Adverse reactions occurred 2.17 ± 8.60 hours (range: 0-48 hours) after mAb infusion, with reactions to casirivimab-imdevimab (4.81 ± 12.98 hours) occurring later compared to the other mAbs on average.

Survey responses

A total of 366 patients (11.3% response rate) were reached via phone call and consented to complete the survey. There were no significant differences between patients who did and did not respond to the survey. The responses are recorded in Table 5.

**Table 5:** Patient survey responses and symptom improvement

	Bamlanivimab-Etesevimab (N=78)	Bebtelovimab (N=78)	Casirivimab-Imdevimab (N=181)	Sotrovimab (N=29)	Totals (N=366)
Symptom Improvement					
Symptoms Improved	62 (79.5%)	65 (83.3%)	144 (79.6%)	18 (62.1%)	289 (79.0%)
No Change	16 (20.5%)	13 (16.7%)	31 (17.1%)	10 (34.5%)	70 (19.1%)
Symptoms Worsened	0 (0.0%)	0 (0.0%)	6 (3.3%)	1 (3.5%)	7 (1.9%)
Improved Symptoms					
Fever	13 (16.7%)	15 (19.2%)	46 (25.4%)	2 (6.9%)	75 (20.5%)
Cough	20 (25.6%)	26 (33.3%)	40 (22.1%)	5 (17.2%)	91 (24.9%)
Fatigue	17 (21.8%)	22 (28.2%)	39 (21.6%)	7 (24.1%)	85 (23.2%)
Myalgia	6 (7.7%)	16 (20.5%)	41 (22.7%)	4 (13.8%)	67 (18.3%)
Shortness of Breath	9 (11.5%)	7 (9.0%)	18 (9.9%)	1 (3.5%)	35 (9.6%)
Headaches	7 (9.0%)	7 (9.0%)	24 (13.3%)	0 (0.0%)	38 (10.4%)
Flu-Like Symptoms	10 (12.8%)	0 (0.0%)	2 (1.1%)	0 (0.0%)	12 (3.3%)
Nausea	3 (3.9%)	11 (14.1%)	12 (6.6%)	0 (0.0%)	26 (7.1%)
Congestion	3 (3.9%)	12 (15.4%)	7 (3.9%)	6 (20.7%)	28 (7.7%)
Sore Throat	0 (0.0%)	9 (11.5%)	10 (5.5%)	2 (6.9%)	21 (5.7%)
Other	14 (17.9%)	6 (7.7%)	24 (13.3%)	0 (0.0%)	34 (9.3%)
Mean Time to Symptom Improvement (Hours)	70.1	46.6	49.0	55.1	53.3
Adverse Effects					
Yes	3 (3.9%)	5 (6.4%)	32 (17.7%)	2 (6.9%)	42 (11.5%)
No	75 (96.2%)	73 (93.6%)	149 (82.3%)	27 (93.1%)	324 (88.5%)

**Figure 1:**
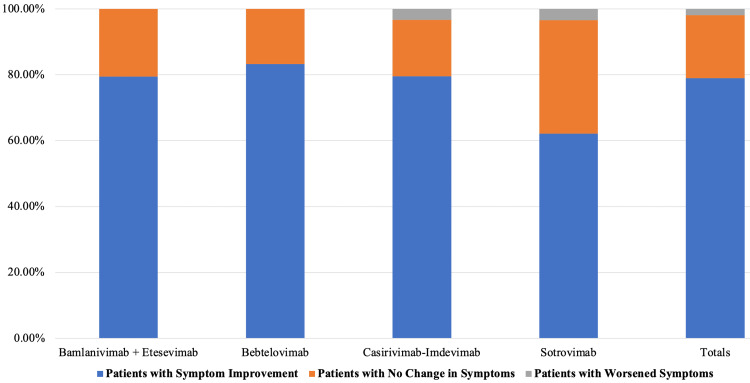
The impact of MAb treatments on SARS-CoV-2 symptoms mAb: monoclonal antibody

Two hundred eighty-nine participants (79.0%) reported symptom improvement following mAb administration, with 70 (19.1%) reporting no change in symptoms and seven (1.9%) reporting a worsening of SARS-CoV-2 symptoms (Figure 1).

There was no significant difference in symptom improvement between the mAbs (p = 0.139). The symptoms that were most improved included cough (24.9%), fatigue (23.2%), and fever (20.5%). The mean time to symptom improvement post-mAb administration was 53.25 ± 72.57 hours, and only 11.5% of survey respondents reported feeling any adverse effects from the mAb infusion.

## Discussion

The main goal of this study is to describe how mAb affects hospitalization rates, hospital length of stay, and mortality rates when administered within 10 days of the SARS-CoV-2 infection. Recent reports have published data indicating mAbs have a role in the early treatment of mild to moderate cases of SARS-CoV-2 to prevent the development of more severe disease [7,11]. However, little data is available regarding patient outcomes following mAb therapy for SARS-CoV-2. This study reports on the use of mAbs in patients presenting to the ED with symptoms of SARS-CoV-2 and patient outcomes, including hospitalization rate, mortality rate, and repeat ED visit(s) following mAb infusion. Additional data included subjective symptom improvement and adverse effects following mAb infusion.

Patients presented to the ED within a nearly two-year period from June 2020 to April 2022 and received different mAbs based on the date each mAb became available. Amongst the patients receiving each mAb, there were coincidental yet significant differences in age, BMI, past tobacco use, race, vaccination status, and select comorbidities, including a history of cancer, diabetes, hypertension, hyperlipidemia, coronary artery disease, a history of stroke, immunodeficiency, and systemic inflammatory disease. Likewise, the patients receiving different mAbs had significant differences in presenting symptoms. This was likely due to the time during the pandemic in which each was administered and the SARS-CoV-2 strains predominant at the time. While these differences limit comparisons between mAbs, there is pertinent data regarding the efficacy of mAbs. Patients treated with different mAbs had significantly different hospitalization rates, between 0.1% and 2.1%. While there is limited value in comparing hospitalization rates between studies, the rates reported in this study are much lower than rates reported earlier in the pandemic, at around 13.3% to 16.1% in 2019 and 2020 [25,26]. 

The mortality rate reported in this study is lower than the reported case fatality rate of 1.1% in the United States as of March 4, 2023, though the strength of the comparison is limited due to population and time differences [27]. Among the mAbs, there were no significant differences in mortality rates between the mAbs, with rates between 0.0% and 0.6% (p = 0.158). This suggests that even though mAbs may have varying efficacy in preventing hospitalization and the incidence of adverse reactions, the four mAbs included in the study were similarly effective in preventing death due to SARS-CoV-2. The mean LOS in this study is lower than the mean LOS reported by Chiam et al., a study conducted in 2020 [28]. However, the LOS reported in this study cannot be attributed to mAb use rather than the timing of the pandemic. The adverse events in this study are similar to those in other studies, though past studies found lower adverse event rates of around 1.9% for bamlanivimab-etesevimab and around 1% for casirivimab-imdevimab [10,23]. The most common adverse events were dizziness or lightheadedness, allergic reactions, nausea or vomiting, and mild hypotension, similar to past investigations of mAb adverse effects [10,23,29,30]. 

The low hospitalization rates, mortality, and repeat ED visits reported in this study are positive findings. Even without a direct comparison group, the patient outcomes reported in this study are promising and show some benefits in mAb use for SARS-CoV-2, though precise quantification of that benefit could not be obtained via the study methods. In some patients who would otherwise be hospitalized, mAb treatments may aid in alleviating symptoms and allow outpatient management, supported by the lower hospitalization rate and LOS post-mAb infusion compared to previous studies not utilizing mAb treatments. The mAbs investigated in this study were shown to have some clinical utility; however, the mutating nature of SARS-CoV-2 requires constant development of new mAb formulations to address new viral strains. The rapid rate of mutation also means that rates of mAb development can lag behind shifts in predominant viral mutations, creating challenges for viral genetic testing and the administration of the correct mAb treatments for specific viral strains. Regardless of clinical efficacy, these obstacles are major limiting factors for mAb use, particularly in areas without testing.

Limitations

This study has several limitations. One limitation is the absence of a control group, as all patients who qualified and consented to mAb infusion received the available mAb treatment. As a result, comparisons to other reported SARS-CoV-2 hospitalization rates, mortality rates, and repeat ED visit rates are limited by the differences in patient populations by region, SARS-CoV-2 strain, past exposure to SARS-CoV-2, vaccination rates, and patient follow-up. The retrospective study design at a single regional hospital also limits the generalizability of the findings. Another limitation is the low survey response rate (11.3%), likely due to the patient population being surveyed and the method of obtaining information regarding patient-reported symptom improvements and adverse effects via phone surveys. Despite the low response rate, the survey data collected still provides crucial information on how the mAb treatment affected the course of each respondent’s illness. The study methodology also precluded our ability to evaluate whether outpatient therapies such as nirmatrelvir/ritonavir, molnupiravir, or remdesivir impacted outcomes for some patients.

## Conclusions

Multiple monoclonal antibodies are used as an adjunct treatment for SARS-CoV-2 and other viral illnesses. This study shows a low rate of hospitalization and mortality following mAb infusion in patients with SARS-CoV-2. Patients reported a high degree of symptom improvement and a low rate of adverse reactions, with no severe adverse reactions. While mAbs are not effective as monotherapy and may only supplement additional treatments, this study shows the potential benefits of including a mAb infusion as part of a SARS-CoV-2 treatment plan.
